# Technical note: Design, development and validation of an automated gas monitoring equipment for measurement of the dynamics of microbial fermentation

**DOI:** 10.1016/j.mex.2022.101641

**Published:** 2022-02-22

**Authors:** Seyed Mohammad Hadi Rahavi, Farhad Ahmadi, Ahmad Vahid, Hamidreza Moinoddini, Mostafa Ghayour, Franco Tagliapietra

**Affiliations:** a Department of Mechanical Engineering, Islamic Azad University, Khomeinishahr Branch, Isfahan, Iran; b Research Institute of Eco-friendly Livestock Science, Institute of GreenBio Science Technology, Seoul National University, Pyeongchang 25354, Korea; c Department of Electrical Engineering, Institute of Higher Education, Isfahan, Khomeinishahr, Isfahan, Iran; d Department of Animal Science, Isfahan University of Technology, Isfahan, Iran; e Department of Agronomy, Food, Natural Resources, Animals and Environment (DAFNAE), University of Padova, Viale dell'Università 16, 35020, Legnaro (PD), Italy

**Keywords:** Automated equipment, Digital pressure gauge, Fermentation kinetic, Gas production

## Abstract

The present technical note describes design, development and validation of an automated equipment for measurement of kinetics of gas production during fermentation in glass bottles. The overall repeatability and precision of the developed system was evaluated and compared with the manual gas measurement technique in respect to characterization of the fermentation kinetics of ruminant livestock feeds. Two incubations were carried out, during which the GP of six different feeds was measured with the automated system or manual technique. During a 48-hour incubation period, pressure data were collected at 15-minute intervals using automated equipment, yielding 192 head-space pressure measurements for each bottle. In manual measurement, incubations were performed with the nominal 60-mL serum bottle, and headspace pressure was read using a digital pressure gauge and then released at 2, 4, 6, 8, 12, 16, 24, 36, and 48 hours of incubation. The automated equipment recorded greater GP (+11.5%, over the 48-h incubation) than the manual measurement, and the repeatability and coefficient of repeatability values indicated that the GP data obtained with manual equipment were less repeatable. The automated equipment measures the fermentative GP kinetics with greater precision and repeatability than manual technique.

• An automated batch GP equipment was designed, developed and validated, and a comparison was made with GP data obtained manually using a digital pressure gauge.
• The automated equipment provided more reliable and repeatable data compared with manual measurement.
• The automated equipment is available with lower cost and more functionality.

## Graphical abstract

**Figure:**
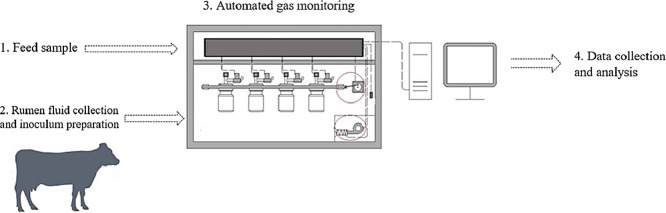

Specifications table

**Table:** 

Subject Area:	Environment Agricultural and Biological Science
More specific subject area:	Ruminant Nutrition
Method name:	Automated Gas Measurement System
Name and reference of original method:	Mauricio, R.M., Mould, F.L., Dhanoa, M.S., Owen, E., Channa, K.S., Theodorou, M.K. 1999. A semi-automated in vitro gas production technique for ruminant feedstuff evaluation. Anim. Feed. Sci. Technol. 79:321−330.
Resource availability:	N.A

## Introduction

Although *in vivo* experiments provide an accurate and reliable estimate of rumen degradability of feedstuffs, they demand substantial labor force, feed, and time, making them unsuitable for large-scale feed evaluation studies [1,^2^,9]. *In situ* techniques were developed to estimate the potential degradation rate of feeds in the rumen [5,18]. However, they have some drawbacks such as high costs of maintaining surgically-fistulated animals, non-applicability of the technique to all feeds, and limitations to simultaneous evaluation of a large number of feed samples [1,12].

Among the *in vitro* techniques, the GP technique allows for the simultaneous determination of the feeding value of a large number of feeds [8,22]. Several GP measurement systems have been developed over the last several decades. Pell and Schofield [20] developed the first automated equipment that measured head-space gas pressure in real time. This equipment is incapable of releasing accumulated pressure during fermentation, which may disrupt microbial activity and, thus affect the rate and extent of fermentability [23,26]. Theodorou et al [26] developed a simple GP system using an electronic measuring procedure and is still a widely-used gas measurement system worldwide. However, it requires the visual reading of volume values, does not provide the simultaneous recordings of all fermentation bottles, and temperature fluctuations may occur while recording GP volumes. Moreover, it is tiresome, labor-intensive, and therefore subject to poor repeatability [11,12]. Mauricio et al [12] developed a semi-automated gas measurement system that still required the hand-held insertion of pressure transducer into each fermentation bottle. Recently, the market has seen the introduction of fully automated GP equipment, which allows for the wireless measurement of GP with a high degree of accuracy (Ankom^RF^ Gas Production System, Ankom Technology Corp., Fairport, NY) [3]. However, the sophistication and high cost of this equipment prevents it from being routinely used in many research laboratories.

Cattani et al [6] compared total GP measurements from closed versus vented bottles, and found that venting fermentation bottles at a low-pressure threshold allows for a reliable measurement of total GP and methane production. The authors proposed that frequent gas venting prevents pressure buildup and partial CO_2_ dissolution, which leads to an underestimation of total GP. However, the current systems based on manual or semi-automated GP measurements do not permit gas venting when the threshold pressure is reached. Therefore, the current study aimed to develop a low-cost, simple-to-use, fully-automated GP system to aid in the study of the microbial fermentative kinetics, and then to compare the repeatability and accuracy of GP recorded with automated versus manual system.

## Materials and methods

### Preparation and chemical composition of experimental feeds

Alfalfa hay, wheat straw, corn silage, barley grain, corn grain, and a total mixed ration (TMR) containing the following ingredients (g/kg): 220 alfalfa hay, 344 corn silage, 236 barley grain, 59 soybean meal, 46 rice bran, 26 fishmeal, 23 fat supplement, and 46 supplements (calcium carbonate, salt, di-calcium phosphate, bicarbonate sodium, magnesium oxide and vitamin-mineral premix), were selected to cover a wide range of chemical composition and degradation rate in the rumen. Prior to chemical analysis, feed samples (approximately 0.5 kg) were milled to pass through a 1.0-mm sieve (Wiley mill, Arthur H. Thomas Co., Philadelphia, PA). Standard methods described by the Association of Official Analytical Chemists [4] were adopted for measurement of dry matter (DM), crude protein (CP), ether extract (EE), and ash. Contents of ash-corrected neutral detergent fiber (with heat-stable α-amylase, 100 µL per 0.50 g of sample; number A3306; Sigma Chemical Co., St. Louis, MO) and acid detergent fiber (ADFom) were measured using an Ankom^220^ Fiber Analyzer (Ankom Technology Corp., Macedon, NY, USA) according to Van Soest et al [27]. The chemical composition of each feed sample is presented in Table 1.

**Table 1:** Chemical composition (g/kg DM) of experimental feeds^a^.

Feeds	DM	CP	EE	NDFom	ADFom	Ash	NFC
Alfalfa hay	924 ± 7.2	182 ± 2.2	24 ± 1.2	318 ± 6.7	242 ± 6.3	118 ± 1.6	358 ± 7.2
Wheat straw	894 ± 5.4	42 ± 1.8	22 ± 0.9	794 ± 7.9	576 ± 6.8	123 ± 1.4	19 ± 1.3
Corn silage	276 ± 8.1	74 ± 1.3	38 ± 1.1	465 ± 6.5	311 ± 8.2	79 ± 0.9	344 ± 7.8
Barley grain	931 ± 7.4	89 ± 1.7	49 ± 1.9	292 ± 5.1	113 ± 4.3	39 ± 1.2	531 ± 6.9
Corn grain	884 ± 6.5	83 ± 2.0	35 ± 1.0	129 ± 4.3	58 ± 3.7	26 ± 0.8	727 ± 9.4
Total mixed ration	578 ± 9.2	165 ± 2.3	39 ± 2.3	328 ± 8.2	193 ± 7.8	91 ± 1.5	377 ± 10.0

CP = crude protein; EE = ether extract; NDFom = ash-corrected neutral detergent fiber; ADFom = ash-corrected acid detergent fiber.

Non-fibrous carbohydrates (NFC), calculated as 100 – [NDFom + CP + EE + ash].

a Data are the mean of 4 replications ± standard deviation

### Experimental design

The representative samples of each feed were divided into 20 sub-samples―16 for incubation and the remaining 4 for chemical composition analysis. The experimental design for each measurement technique (automated or manual) was as follows: two incubation runs × six feeds × four replications (per run per feed), giving a total of 8 measurements per each feed, plus 12 blank bottles (only buffered rumen fluid). The amount of gas produced in blank bottles within each run was averaged and subtracted from the GP of feed samples.

### Description of the automated equipment

**Fig. 1:**
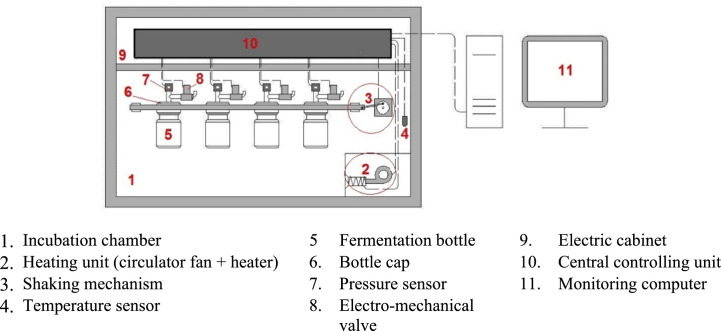
Schematic of automated gas monitoring equipment.

The schematic representation of the automated GP equipment is illustrated in Fig. 1. Supplementary Fig. S1 also illustrates pictures of the automated system. The apparatus included three arrays of 12 glass bottles with a total volume capacity of 140 mL (Glassco Bottle, Laboratory; Part No. 274.202.01). Each polypropylene plug-seal screw cap (Part No. 275.205.01; blue color screw cap GL-45) was fitted with a 1.5-mm polypropylene pouring ring (Part No. 275.205.02) and an O-ring washer, ensuring that each fermentation bottle was strictly air-tight during the incubation period. The reciprocating mechanism was provided by a crank mechanism to convert rotation to linear movement. The amplitude of the movement was set to 50 mm, with an adjustable speed of 0−80 rpm. The mechanism was driven with a three-phase induction motor and worm gearbox (reduction rate of 20:1). Temperature control was provided using a solid-state relay (SR1-122, Autonics, South Korea), two fixed-speed circulating fans, two 1000-watt heating elements placed in the bottom corners of the incubation chamber, and a 3-wire PT100 temperature sensor (range = −40 to +180°C) that measured the internal temperature of the incubation chamber. A 30-mm expanded polystyrene sheet was placed between the outer (16-mm medium-density fiberboard panels) and inner wall (1-mm aluminum sheet) of the incubation chamber to provide thermal insulation. An electro-mechanical valve (operation pressure = 0–5 bar; CEME 5523, China) and a 5-V DC-operated high-sensitivity pressure sensor (MPX5100GP, Case 867B; Freescale Semiconductor Inc.; Texas, USA; pressure range = 0−100 kPa; accuracy = ± 2.5% of measured value; response time = 1.0 millisecond) were installed on each screw cap that had previously been affixed to the base plate. Using a DVP-14SS211T programmable logic controller, appropriate modules (Delta Electronics Inc., Taiwan) and a monitoring and controlling software, the head-space pressure was recorded with 15-min intervals, resulting in 192 data points for each bottle during a 48-h incubation. Because of the negative impact of head-space overpressure on the rate of GP fermentation, headspace gas build-up must be vented on a regular basis [23,26]. This effect was minimized through releasing head-space gasses at 15-min intervals immediately after each gas pressure measurement. This ensured that accumulated pressure never exceeded the critical threshold, especially during the rapid phase of gas release. Connections between Luer adapters and bottle cap, or the Luer and pressure sensor or valve, were secured and leak-proofed with a sealant adhesive. Each pressure sensor/electromechanical valve assembly was inspected for pressure decline (leak) or permeability to gases by injecting 90 kPa of CO_2_ or CH_4_ into the sensors, and then the overnight monitoring of pressure [1]. The system was controlled through a customized software written in the C# programming language (Version 5.0; Microsoft Corp., 2013). The hardware was linked to a computer via an RS-232 serial port, which monitored and recorded the temperature and pressure dataset on an Excel spreadsheet (.xlsx, Microsoft Corp. 2013). Both the present and cumulative head-space pressures of individual bottles were monitored during the recording process, allowing for immediate feedback, process control, and graphical visualization of the real-time fermentation progress throughout the process.

### Calibration of pressure sensors

Using a 3-way connection, each sensor was separately connected to a test gauge (Model: DG 60; Serial No. 140725010, Indumart Inc., Canada) and an adjustable pressure source. Individual sensor calibration was accomplished by injecting pressure and reading the corresponding digital values of 0, 5, 10, 15, 20, kPa incrementally and 20, 15, 10, 5, 0 kPa subtractively. This procedure was repeated twice for each pressure sensor, yielding a calibration dataset containing 720 points for 36 sensors. The 36 conversion equations were derived and entered into the software and the accurate pressure value was achieved.

### Response and leak test

The validity and drift of the sensor response was checked prior to the initiation of each incubation run, as described by Muetzel et al [17]. In brief, each sensor was attached to an incubation bottle containing a known volume of water (60 mL) and air (1–10 mL). Thereafter, the system was operated and the difference between head-space pressure and ambient pressure was recorded, and then compared with the response from the calibration series. The system measured the pressure for a duration of 15–1000 min to detect any pressure drop (leak).

### Rumen fluid preparation

Two intact rumens of sheep (body weight = 55 ± 1.4 kg) were obtained from a slaughterhouse for each incubation run, stored in a warm insulated container, and immediately transferred to the laboratory (within 10 min). According to the slaughterhouse owner, the sheep had been fed a TMR containing 700 g/kg alfalfa hay and 300 g/kg barley grain, with free access to water. The composite ruminal inoculum was prepared according to Mourino et al [16]. The rumen fluid was then mixed with an anaerobic pre-warmed buffer/mineral solution (1:2 v/v; [14]) and bubbled for at least 15 min while being continuously flushed with oxygen-free CO_2_ and constantly stirred on a stirring hot plate. This provided moderate mixing for uniformity (∼ 40 rpm) as well as temperature control (39 ± 0.5°C) to equilibrate the fluid before it was dispensed into the pre-warmed bottles, thereby avoiding the exposure of microorganisms to cold shock and aerobiosis. The color change of resazurin indicator indicated CO_2_ saturation. All pH values measured after termination of the incubation (48 h) were higher than the 6.2 threshold (data not presented), implying that the culture medium maintained its buffering properties [13]. To avoid particle dispersion, prior to dispensing the buffered rumen fluid, feed samples (estimated *a priori* to be ∼ 200 mg of DM) were weighed in the bottles and moistened with distilled water (2.0 mL), which was subtracted from the media [25]. The bottles were filled with buffered rumen fluid at random and placed in the incubator.

### Automated GP measurement

After the rumen fluid (60 mL) was dispensed, the bottles were flushed with CO_2_ for 10 seconds before being immediately capped onto each affixed screw cap inside the pre-warmed incubating chamber (39 ± 0.5°C). After each pressure reading, the current pressure was cumulated with the previous readings and the present cumulative pressure was recorded. The starting time was set to zero, and the recording was stopped after 48 h.

### Manual GP measurement

Manual GP measurement was carried out in accordance with Weimer et al [28]. Pressure accumulating in the headspace of volume-calibrated serum bottle (nominal volume 60 mL) with respect to the atmospheric pressure was measured using a battery-powered digital pressure gauge (Model: DG60; Serial No. 140725010, Indumart Inc., Canada; accuracy = 0.25 % of full scale), which was calibrated to read pressure unit (kPa). The buffered rumen fluid was dispensed into the serum bottles, which were immediately flushed with CO_2_, closed with a 14-mm rubber septum, secured with an aluminum crimp seal, slightly agitated, and then placed in a shaking water bath (Memmert, Model WNE 10, Schwabach, Germany). The water bath was set to 50 back-and-forth movements per minute at temperature of 39 ± 0.5°C. The bottom of the pressure gauge was fitted with a two-way metal valve, with the first outlet connected to a disposable hypodermic syringe needle (23 gauge), which was replaced after every six stopper penetrations. The second outlet was connected to an open-closed electromechanical micro-valve (CEME 5523, China), which allowed the accumulated head-space gas pressure to return to atmospheric pressure. The connections were checked to ensure they were gas-tight before the measurement. The head-space gas pressure of each bottle was recorded and immediately depressurized using an electro-mechanical valve at pre-determined incubation times (2, 4, 6, 8, 12, 16, 24, 36, and 48 h), as indicated by a zero-pressure reading on the LCD display. Because only a few bottles were removed from the incubator at a time, it was assumed that the temperature of the head-space gas remained constant throughout the measurement period.

### Computations

Pressures (kPa) were converted into unit of volume (mL) using the ideal gas law according to Eq. (1) [24]:

(1) $$\text{GP}=\frac{\left[\left(P1+Patm\right)\times V0\right]}{Patm}$$

where, *P*_1_ is the cumulated pressure (kPa) in the bottle headspace and *P*_atm_ is the atmospheric pressure read by the equipment at the beginning of the study; *V*_o_ is the bottle head-space volume. The cumulative GP data were fitted with MATLAB software (version 8.1.0.604 (R2013a), The MathWorks, Inc., Natick, Massachusetts) to the following non-linear Eq. (2) [19]:

(2) $$P=B\left(1-\text{ex}p^{-c(t-L)}\right),$$

where, *P* = GP (mL) at time *t, B* = asymptotic GP (mL/g DM), *c* = GP rate constant (h^−1^), *t* = incubation time (h) and *L* = lag time (h).

### Repeatability test and statistical analysis

The repeatability (RT) was defined as the value below which the absolute difference between two single measures obtained using the same techniques and under the same conditions (same incubation run, same feed, etc.) is expected with a 95% confidence [6]. The values of RT and coefficient of repeatability (RT%) were calculated according to the following Eq. 3 and 4 [10].

(3) $$\text{RT}=2\sqrt{2\sigma_{e}^{2}},$$

where σ^2^_e_ is the residual variance.

(4) $$\text{RT}\%\frac{{\sigma^{2}}_{R}+{\sigma^{2}}_{F}+{\sigma^{2}}_{R\times F}}{{\sigma^{2}}_{R}+{\sigma^{2}}_{F}+{\sigma^{2}}_{R\times F}+{\sigma^{2}}_{e}}\times 100,$$

where σ^2^_R_ = the variance among 2 runs, σ^2^_F_ = variance among feeds, and σ^2^_R × F_ = run-by-feed variance. The components of variance for each factor were estimated using the restricted maximum likelihood method [24].

Incubations and data analysis were carried out using a 2 × 6 factorial design, with the six feeds tested simultaneously in each of two consecutive incubations using the two techniques (automated or manual measurement). The data were analyzed using the Proc Mixed of SAS (SAS Institute, 2002), with the following model: Y_ijk_ = µ + T_i_ + F_j_ + (T × F)_ij_ + R_k_ + e_ijk_, where Y_ijk_ = the response variable; µ = the overall mean; *T =* measurement technique (i = 2; automated or manual); F = the effect of feed (j = 6); (T × F)_ij_ = the interaction effect between measurement technique i and feed j; R_k_ = the effect of incubation run as a random factor (R = 2) and e_ijk_ = the residual random error. Mean comparisons were performed using Tukey's multiple range test, and significance was declared at *P* < 0.05.

## Results and discussion

Table 2 reports the cumulative GP of the six feeds measured with an automated system or manual technique, expressed in mL/g of incubated DM. On average, the automated equipment recorded higher gas volumes than the manual system [11.5, 13.2 and 11.5% higher volumes of GP following 24, 36 and 48 h of incubation, respectively; *P* < 0.01]. In support, Cattani et al [6] discovered that fermentation in closed bottles resulted in a significant underestimation of total GP (24 h) than fermentation in vented bottles (an average of 18%; after 24-h incubation). The authors also stated that the magnitude of GP difference was clearly visible for highly fermentable feeds such as corn grain (−56 mL of gas/g of incubated DM) and sugar beet pulp (−54 mL of gas/g of incubated DM).

**Table 2:** Effect of gas measurement technique, feed, and their interaction on cumulative gas production (GP, mL/g of incubated DM) after 6, 12, 24, and 48 h of incubation^1^.

Items	GP_6_	GP_12_	GP_24_	GP_48_
Technique				
Automated	80	111	145	185
Manual	75	104	130	166
SEM	1.4	1.6	2.7	3.5
Feed				
Alfalfa hay	71	103	126	153
Wheat straw	20	51	77	115
Corn silage	70	96	123	159
Barley grain	94	124	162	218
Corn grain	115	146	183	224
Total mixed ration	97	125	153	183
SEM	2.5	2.8	3.9	4.9
*P*-value				
Feed (F)	<0.01	<0.01	<0.01	<0.01
Technique (T)	0.02	<0.01	<0.01	<0.01
Feed-by-technique interaction (T **×** F)	0.26	0.17	0.95	0.92
Manual				
RT^2^	33.9	38.5	44.9	53.3
RT%^3^	87.3	82.2	83.7	81.2
Automated				
RT	24.3	27.1	36.3	47.2
RT%	94.0	93.3	89.7	87.0

1 Each value is the mean of 12 observations.

2 Repeatability (RT) was computed as 2$\sqrt{2\sigma^{2}e}$, where σ^2^_e_ is the residual variance.

3 Coefficient of repeatability (RT%) =$\frac{{\sigma^{2}}_{R}+{\sigma^{2}}_{F}+{\sigma^{2}}_{R\times F}}{{\sigma^{2}}_{R}+{\sigma^{2}}_{F}+{\sigma^{2}}_{R\times F}+{\sigma^{2}}_{e}}\times 100$ where, σ^2^_R_ is the variance among two incubation runs, σ^2^_F_ = variance among feeds, and σ^2^_R × F_ = incubation run × feed variance [10].

**Fig. 2:**
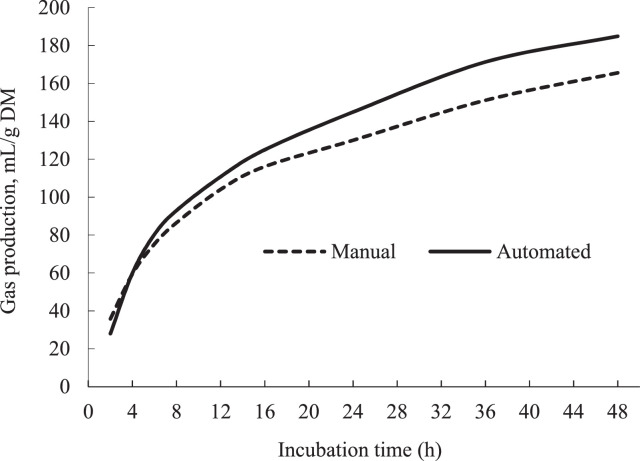
Gas production measurements determined with automated system (solid lines) or manual technique (dotted lines) where, in two incubation runs six feeds were incubated in four replications for 48 h.

As presented in Fig. 2, the magnitude of difference in total GP was negligible during the first hours of fermentation, followed by more pronounced differences as fermentation progressed; on average +19 mL of gas/g of incubated DM was produced with the automated equipment. In agreement, Cattani et al [6] reported the same trend and reasoned that the head-space pressure is low during the first hours of fermentation, but as fermentation proceeds, the progressive increase in the head-space gas pressure leads to the partial solubilization of CO_2_ into the buffered rumen fluid, resulting in an underestimation of actual GP. This implies that venting frequency is important in obtaining actual GP estimates, emphasizing the importance of using an automated system with adjustable venting frequency for more accurate GP measurements.

Although RT values were higher with GP data obtained by manual measurement than with automated equipment, the RT% trend was the opposite. For example, the RT values for GP_48_ were 53.3 and 47.2 mL of gas/g DM incubated, for manual and automated measurement, corresponding to RT of 81.2 and 87.0%, respectively (Table 2). This implies that GP measurements using automated equipment have higher repeatability and accuracy than manual measurements [6]. As expected, there was a significant difference in GP values between experimental feeds (*P* < 0.01), implying that the two measurement techniques were successful in ranking the various feeds in terms of their GP potential. For example, the GP during a 48-h incubation from highest to lowest was in the order of total mixed ration, corn grain, barley grain, corn silage, alfalfa hay, and wheat straw. The effect of measurement technique (*T*) on the gas volumes at the different incubation times was significant (*P* < 0.05; Table 2). This suggests that the feeds were ranked differently by the two measurement techniques based on their GP potential. The interaction of feed × measurement technique was not significant at any incubation times studied (*P* > 0.05).

**Fig. 3:**
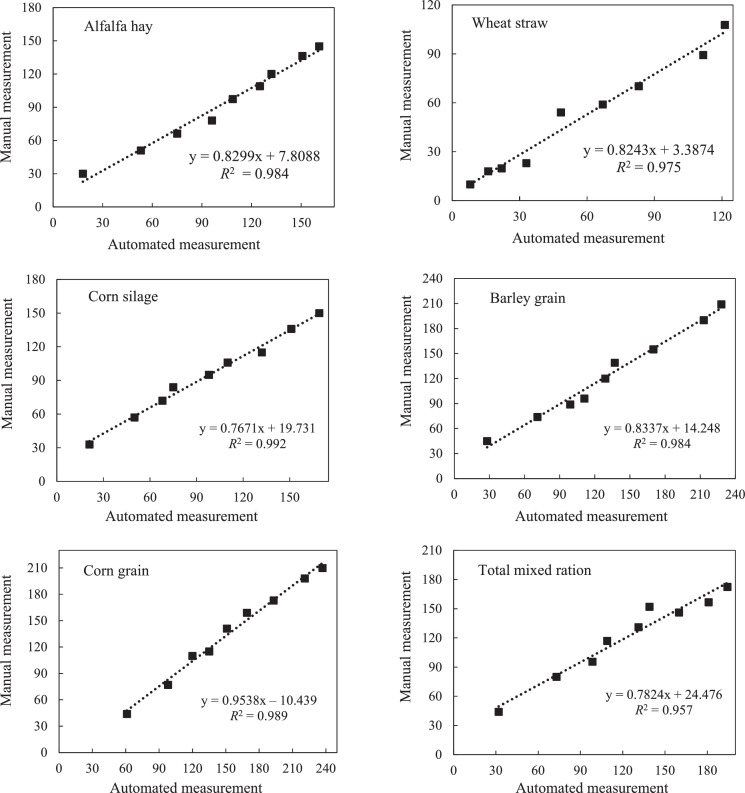
Relationship between total gas production (mL/g DM) at different incubation times [2, 4, 6, 8, 12, 16, 24, 36, and 48 h] for each feed ingredient measured with automated system or manual technique.

Fig. 3 illustrates the GP estimates calculated using interpolation at different incubation times for each feed, with pressure measurements obtained with the automated equipment versus those obtained manually. Minor differences existed between the two measurement methods, and when gas volumes were recorded with automated equipment, GP values were higher than those recorded manually, especially during the final times of incubation. Deviations from the equality line were highest for TMR and wheat straw, but negligible differences were identified with other feeds.

**Fig. 4:**
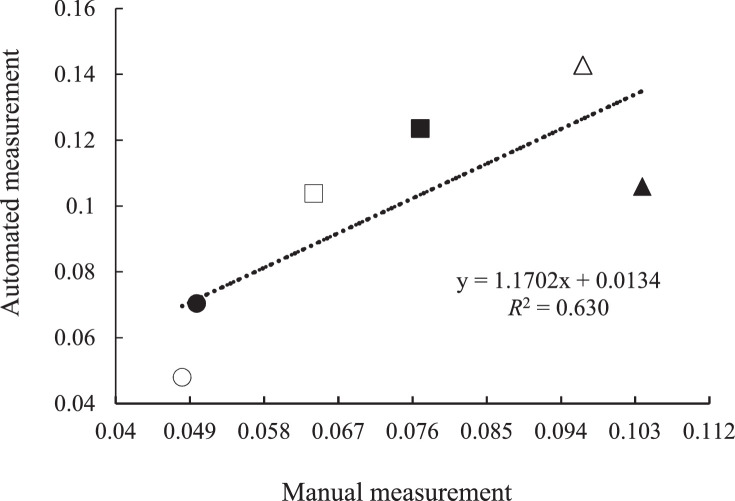
Relationship between gas production rate constant (h^−1^) of six experimental feeds (○ wheat straw; ● barley grain; □ corn silage; ■ corn grain; ∆ total mixed ration; ▲ alfalfa hay) measured with automated or manual system.

A weak relationship (*R*^2^ = 0.63) existed between GP rate constant of feed samples measured with automated and manual system (Fig. 4). It appears that the GP kinetic estimation through manual measurement does not accurately reflect the actual GP and, thus, may not be a reliable method to rank the feeding value of feeds. This observation is supported by the higher total GP recording with the automated GP equipment versus manual system.

### Comparison with other GP systems

Compared with the manual [26] or semi-automated GP systems [12], the automated GP equipment provides simultaneous GP measurement of all bottles, which improves the accuracy and reliability of data acquisition. Moreover, the automated GP equipment could be programmed to record head-space pressure at predetermined time intervals throughout the incubation, resulting in a high density of data points during the first hours of incubation, when rumen microbial fermentation is most active. The automated equipment has some advantages over ventless systems, such as the system developed by Pell and Schofield [20], which do not allow for the correction of variations in atmospheric pressure with blank bottles, and thus analysis of residual gases accumulating in the headspace. Moreover, if the accumulated head-space gas pressure exceeds the critical threshold, a portion of CO_2_ might dissolve in the culture medium, which underestimates GP values [6,23], lowers the medium pH, and perturbs microbial activity and fermentative pathways [15,29]. For venting head-space gas accumulation, the software offers two options: venting by pre-defining a sequence of time points or venting by fixing a defined head-space pressure threshold. The latter option may be preferred in some experiments because, if venting is triggered by a pressure threshold rather than time, it ensures that the pressure in the bottles never exceeds the 4.5 kPa threshold, which has been linked to increased CO_2_ solubility at high pressures (Davies et al., 2000; Calabrò et al., 2005). Cattani et al [6] demonstrated that frequent gas venting during incubation allows for a more reliable measurement of total GP. As a result, with this software option, the venting frequency relative to the amount of fermentable organic matter is carefully adjusted to avoid head-space gas over-pressures, thereby minimizing CO_2_ solubility.

The current equipment allows the use of culture bottles with varying volume capacities but the same diameter opening, resulting in a different head-space to culture medium ratio, which is known to have a significant impact on head-space gas over-pressures during microbial fermentation [17,21]. The current equipment costs about US$ 12,000 for a complete 36-bottle unit, which is much less expensive than the automated system developed by Cone et al [7], which costs about US$ 15,000 for a 12-bottle unit [1]. A complete set of 36 modules for the ANKOM^RF^ Gas Production System was estimated to cost US$ 20,000 (retrieved on January 15, 2021 from https://www.ankom.com/product-catalog/ankom-rf-gas-production-system). A shaking water bath must be added to this, which is not required with the current equipment.

## Conclusions

Greater gas was recorded with the automated equipment relative to manual measurement. The repeatability and coefficient of repeatability values from GP measurements suggested that the automated equipment produced more accurate and reliable GP data than manual measurement with a pressure gauge, perhaps because of the frequent gas venting during the fermentation, which avoids excess pressure build-up in headspace. Greater sensitivity and less labor required to control the fermentation process in the automated system demonstrate the importance of this system for the easy and routine characterization of ruminant feeds. In comparison to the more complex equipment, current system has advantages such as low initial production costs, ease of maintenance, and commercial availability of its components.

## Declaration of Competing Interest

None

## Appendix Supplementary materials

Image, application 1
