# Supplementary figures and images for: Technical note: Design, development and validation of an automated gas monitoring equipment for measurement of the dynamics of microbial fermentation
Source: MethodsX. 2022 Feb 22;9:101641. doi: 10.1016/j.mex.2022.101641 (PMC8892151; doi:10.1016/j.mex.2022.101641)

| 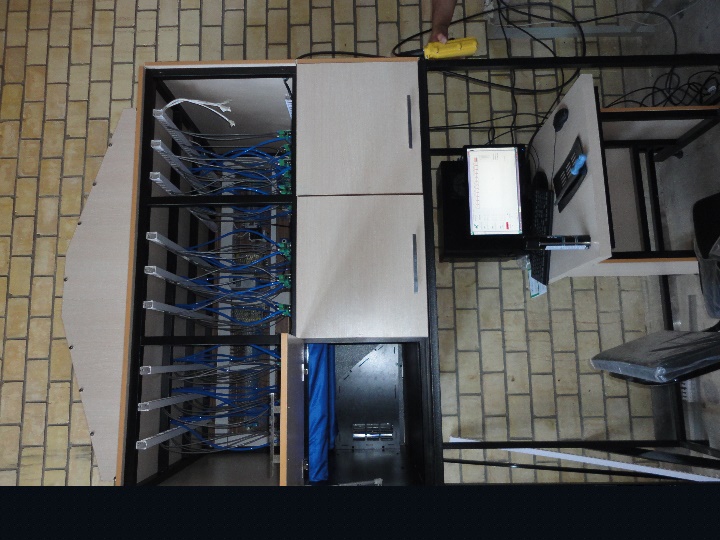 |
| --- |
|  |
| 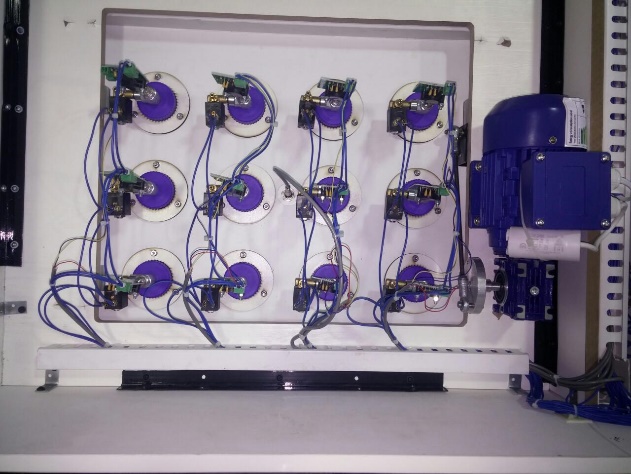 |
|  |
| 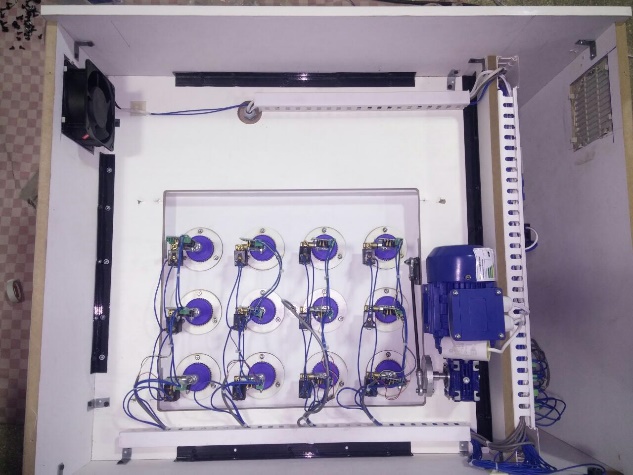 |

**Supplementary Figure S1**. Photographic illustration of the automated gas production system.

Supplement: Supplementary file 1 [file mmc1.docx]
